# A Peculiar Fracture of the Lower Jaw

**Published:** 1883-11

**Authors:** 


### A Peculiar Fracture of the Lower Jaw.

A man falling from a height of fifteen feet was caught under the lower jaw by a beam. The only appreciable injury was a gaping cut which was closed with wire sutures by Dr. Thomas M. Watt, who reports the case in the *Brit. Med. Jour.*, September 15, 1883. Two weeks subsequently, when at tea, he heard a sudden snap in his jaw, and felt as if he had been suddenly struck a hard blow. A fracture was now evident. Dr. W. supposes that the bone was fractured at the time of the original accident, but the fragments were held together by its integuments and soft parts. Suddenly, under no special strain, the connections snapped and the fracture became apparent. No special treatment was adopted, save the abstention from *chewing*, and a cure is supposed to have resulted, as the man did not turn up again.

